# Unveiling Lyme Neuroborreliosis in the Absence of Dermatological Symptoms

**DOI:** 10.7759/cureus.76473

**Published:** 2024-12-27

**Authors:** Lanah Almatroud, Alyaa Saleh, Jibran A Sheikh

**Affiliations:** 1 College of Human Medicine, Michigan State University, East Lansing, USA; 2 College of Medicine, Central Michigan University, Saginaw, USA; 3 Internal Medicine, Central Michigan University, Saginaw, USA

**Keywords:** borrelia burgdorferi infection, cervical radiculitis, lyme borreliosis, lyme's disease, neuro inflammation, neurologic symptoms, tick-bite

## Abstract

Lyme neuroborreliosis can present with isolated neurological manifestations, posing diagnostic challenges, especially in the absence of hallmark dermatological symptoms like erythema migrans. This case highlights a patient with isolated cervical radiculopathy due to Lyme neuroborreliosis, presenting without systemic features such as fever, arthralgia, or rash. The diagnosis was confirmed through serological testing, with positive findings on the Western blot. Comparisons with reported cases, including isolated urinary retention, bilateral neurosensory hearing loss, and Bannwarth syndrome, reveal the clinical heterogeneity of Lyme neuroborreliosis. Elevated inflammatory markers, tick exposure, and serological findings remain crucial for diagnosis. This case emphasizes the importance of clinical vigilance in endemic areas, as timely recognition and treatment are essential to prevent long-term complications.

## Introduction

Lyme disease (LD) is the most common tick-borne illness in the United States [1]. It is transmitted exclusively by the bites of infected *Ixodes* ticks containing the spirochete *Borrelia burgdorferi* (*B. burgdorferi*), resulting in a condition called Lyme borreliosis [2]. Small mammals such as mice and chipmunks serve as the natural reservoir for *B. burgdorferi*, while *Ixodes* ticks rely primarily on deer as their preferred hosts to complete their life cycle. The initial replication of *B. burgdorferi* occurs at the site of inoculation, spreading systemically after activation of the innate and adaptive immune responses [3]. When it infects the nervous system, it’s referred to as Lyme neuroborreliosis, which can lead to symptoms such as facial nerve palsy/cranial neuropathy, radiculopathy, and meningitis [4].

LD is endemic to the Upper Midwest, mid-Atlantic, and Northeastern regions of the United States [5]. While around 30,000 annual cases of LD are reported in the United States, the actual number of cases is suspected to be significantly higher [3]. Failure to identify and treat LD can result in significant long-term morbidity. In a longitudinal study conducted in Massachusetts, a highly endemic area, untreated LD was associated with various complications, including relapsing arthritis and persistent fatigue lasting up to 15 years [6]. This emphasizes the importance of promptly identifying cases to prevent such debilitating outcomes.

One of the potential early manifestations of LD is radiculopathy, a condition characterized by pain and/or sensory and motor deficits, which occurs due to inflammation or compression of a nerve root [7,8]. While radiculopathy in LD typically presents alongside systemic symptoms such as rash, fever, chills, malaise, and myalgia, cervical radiculopathy as an isolated manifestation of LD is extremely rare. By contrast, more common causes of cervical radiculopathy include disc herniation, spondylosis, trauma, instability, or, in rare instances, tumors [9].

While only 5% of diagnosed LD cases involve cervical radiculopathy [10], our patient uniquely presented with isolated cervical radiculopathy as the sole manifestation of LD.

This rare presentation highlights the importance of maintaining a high index of suspicion for LD, even when the sole presenting symptom is radiculopathy. Our case also illustrates the challenges of distinguishing this presentation from degenerative disc herniation-associated radiculopathy or a “pinched nerve,” which led to an initial misdiagnosis.

## Case presentation

A 43-year-old woman initially presented to the emergency department (ED) with acute onset of severe left-sided neck and back pain accompanied by a burning sensation radiating down her left arm into the fingers. She also reported intermittent paresthesia but denied any red-flag symptoms or systemic complaints. She described a recent history of lifting an 80-pound cat litter box overhead, which preceded two days of worsening symptoms. Upon arrival at the ED, her vital signs were normal, except for an elevated blood pressure of 160/91 mmHg. Physical examination revealed mild tenderness over the left paraspinal region and pain with movement of the left shoulder joint. The Spurling test was negative, and no focal neurological deficits were identified. She was diagnosed with a “pinched nerve” and discharged with a short course of prednisone and muscle relaxants.

Over the subsequent month, the patient presented to the ED three more times for the same symptoms, reporting only brief and partial relief with the prescribed steroid treatment. During one of these visits, a cervical computerized tomography (CT) scan was performed, which was unremarkable. Physical examination at this time showed persistent left paraspinal tenderness and reduced trapezius strength. The patient maintained a full range of motion in the neck and was able to lift both arms without pain during abduction or adduction. Initial laboratory studies showed an elevated erythrocyte sedimentation rate (ESR) of 29 mm/h (normal range: 0-10 mm/h) and a C-reactive protein (CRP) of 20 mg/L (normal range: <9 mg/L) (Table 1). Despite normal imaging and non-specific laboratory findings, an inflammatory arthropathy was suggested, and the patient was again prescribed methylprednisolone.

**Table 1:** Trends in inflammatory marker levels over the course of diagnosis and treatment for Lyme neuroborreliosis

Laboratory tests	At initial presentation	One week post-corticosteroid therapy	Six weeks post-antibiotic treatment
Erythrocyte sedimentation rate (mm/h)	29	68	13
C-reactive protein (mg/L)	20	20.1	<3.00

**Figure 1:**
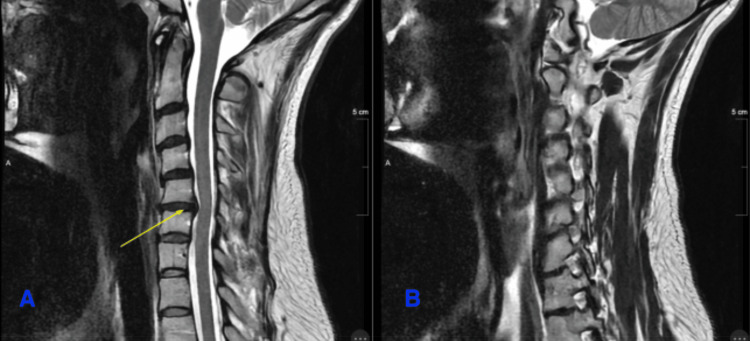
MRI of the cervical spine: T2 sagittal images (A) A moderate disc bulge posteriorly abutting the spinal cord at the C5-C6 level (yellow arrow) without any cord edema. (B) No neuroforaminal compromise at any level.

Approximately two weeks later, the patient returned to the ED with new-onset difficulty chewing and a “locked-jaw” sensation, in addition to her persistent neck, back, and arm pain. On further questioning, she disclosed living in Wisconsin, an endemic area for LD [5], and having a tick bite approximately two months earlier. She recalled that the tick was attached to her thigh for less than 24 hours, and the site developed blistering without erythema. Given this history and the persistence of her symptoms, a cervical MRI and *Borrelia* serology were ordered. MRI findings demonstrated degenerative disc changes with a moderate broad-based disc bulge and central disc herniation at the C5-C6 level, abutting the ventral surface of the spinal cord without evidence of cord compression or neuroforaminal compromise (Figure 1).

*Borrelia*-specific IgG and IgM antibodies were detected in the patient’s serum, with positive findings of three of three IgM and six of 10 IgG bands on Western blot, which, combined with her clinical history and the absence of significant neural foraminal compromise on MRI, confirmed the diagnosis of subacute neuroborreliosis. Follow-up laboratory studies revealed markedly elevated ESR of 68 mm/h and CRP of 20.10 mg/L (Table 1). Additional autoimmune testing, including cyclic citrullinated peptide, rheumatoid factor, and antinuclear antibody panels, was negative, ruling out other inflammatory conditions. Treatment for acute neuroborreliosis was initiated with a 21-day course of doxycycline. 

At her six-week follow-up, the patient reported complete resolution of her symptoms, and repeat laboratory studies demonstrated normalization of ESR and CRP levels (Table 1). 

## Discussion

While erythema migrans occur in approximately 80% of LD cases [6], it is not always present or recognized by the patient, which can delay diagnosis. Additionally, an ESR greater than twice the upper limit of normal is observed in only 24% of LD patients [11], emphasizing the importance of considering LD even when inflammatory markers are not markedly elevated. Our patient’s ESR of 68 mm/h and CRP of 20 mg/L fell within this moderate elevation range, further reinforcing the importance of clinical vigilance in endemic areas. 

The diagnosis of LD relies heavily on clinical suspicion supported by serological testing. For our patient, positive findings of three of three IgM and six of 10 IgG bands on Western blot confirmed the diagnosis. According to CDC guidelines, serological confirmation requires at least two of three IgM bands or five of 10 IgG bands [12], ensuring specificity in differentiating LD from other conditions that mimic its neurological manifestations, such as viral radiculitis, herniated discs, or autoimmune disorders.

The rarity of isolated cervical radiculopathy, as seen in our patient, emphasizes the diagnostic challenges posed by atypical presentations of LD. Isolated cases of Lyme neuroborreliosis can manifest in various forms, often without the hallmark features of LD, such as erythema migrans (a red, expanding rash often resembling a bullseye that typically occurs at the site of a tick bite) [13], arthralgia, or fever. This diagnostic challenge is illustrated by Olivares et al., who reported a 54-year-old male with isolated urinary retention due to acute transverse myelitis secondary to Lyme neuroborreliosis [14]. Like our patient, their case lacked systemic symptoms and presented with a single neurological deficit. However, notable differences, including a significantly higher ESR of 95 mm/h and a prolonged two-month period of urinary symptoms before referral, demonstrate the variable clinical spectrum of LD. These variations highlight the importance of early serological testing and imaging in establishing a diagnosis.

Similarly, Rochd et al. reported an unusual case of isolated Lyme neuroborreliosis presenting as bilateral neurosensory hearing loss [15]. Unlike the neurological deficits in our case, their 23-year-old patient experienced sudden hearing loss, tinnitus, instability, and headache. Pure-tone audiometry confirmed significant bilateral hearing loss, and serologic testing identified *B. burgdorferi* IgM, with Western blot analysis confirming the diagnosis [15]. Despite treatment with corticosteroids and a 21-day course of doxycycline, the patient experienced no symptom resolution and required evaluation for a cochlear implant. The extended two-month period from symptom onset to treatment initiation illustrates the importance of timely recognition and management to prevent irreversible complications.

Further illustrating the heterogeneity of Lyme neuroborreliosis, Diaz and Wesley reported a case involving Bannwarth syndrome with painful meningoradiculitis, facial nerve palsy, and cerebrospinal fluid lymphocytic pleocytosis [16]. Unlike our patient’s localized presentation, their case involved systemic symptoms, including profound transaminitis and weight loss. Despite these differences, both cases benefited from timely medical therapy, underscoring the importance of clinical suspicion in guiding diagnostic and therapeutic decisions. 

Excluding LD as a differential diagnosis is particularly important in isolated neurological presentations. For instance, the Clinical Practice Guidelines for Bell’s Palsy recommend ruling out LD before diagnosing idiopathic Bell’s palsy [17]. This principle extends to radiculopathy, as highlighted in our case. Early identification and treatment of LD are essential to prevent long-term complications, such as cognitive decline, stroke, and other neuropsychiatric conditions [18].

## Conclusions

In Lyme-endemic areas, the differential diagnosis of radiculitis should always include LD, particularly when the presentation is atypical. Radiculitis can be an isolated manifestation of early LD, emphasizing the importance of considering this etiology even in the absence of systemic symptoms. Our case highlights that elevated inflammatory markers, a history of tick exposure, and suggestive serological findings should prompt further investigation. Timely recognition and treatment of Lyme-associated radiculitis are crucial, as they prevent progression to chronic complications and significantly improve patient outcomes. This case adds to the growing body of evidence that Lyme neuroborreliosis can manifest in atypical forms and highlights the need for enhanced diagnostic awareness among clinicians. Future research should focus on understanding the pathophysiology and clinical spectrum of Lyme-associated radiculopathy to improve diagnostic algorithms and patient outcomes.
